# Knowledge, Attitudes and Behaviors on Child Passenger Safety among Expectant Mothers and Parents of Newborns: A Qualitative and Quantitative Approach

**DOI:** 10.1371/journal.pone.0146121

**Published:** 2016-01-06

**Authors:** Xiangxiang Liu, Jingzhen Yang, Xiaojun Chen, Liping Li

**Affiliations:** 1 Injury Prevention Research Center, Medical College of Shantou University, 22 Xin Ling Road, Shantou, 515041, China; 2 Injury Prevention Research and Police Center, The University of Iowa, Iowa City, Iowa, Columbus, Ohio, 43205, United States of America; 3 The First Affiliated Hospital of Shantou University Medical College, Shantou, 515041, China; Beihang University, CHINA

## Abstract

**Objective:**

To investigate the knowledge, attitudes, and intended behaviors about use of child safety seats among parents of newborns and explore expectant mothers’ views and decisions regarding child safety seats use.

**Methods:**

A cross-sectional survey and semi-structured interview were conducted in the maternity departments of two hospitals in China. Parents of newborns were recruited after delivery and surveyed on their knowledge, attitudes and behaviors regarding child safety seats use. Pregnant women were also interviewed to learn about their views and decisions regarding child safety seats use. Both quantitative and qualitative methods were used to analyze the data collected.

**Results:**

Of a total of 242 parents of newborns recruited in the quantitative survey, 202 (83.5%) parents had heard of child safety seats and 149 (61.6%) parents reported they would use child safety seats for their babies. Parents’ knowledge, car ownership, occupation, and income were significantly associated with their decision regarding use of child safety seats. Three themes were identified from the qualitative interview of 30 pregnant women: (1) the pregnant women perceived child passenger safety as important; (2) the car ownership and price and quality of child safety seats were major influencing factors of their decisions on use of child safety seats; and (3) lack of awareness and lack of laws requiring use were perceived to contribute to low use of child safety seats in China.

**Conclusion:**

Lack of knowledge and awareness on child passenger safety were found to be two most important factors associated with low use of child safety seats. Effective interventions are urgently needed to improve parents’ knowledge before laws are enacted and implemented.

## Introduction

Motor vehicle crash related injuries and deaths among children is becoming an increasing global public health problem[1]. Road traffic crashes have become the second leading cause of injuries and deaths for children ages 5 to 14, second only to infectious disease[2]. A report from World Health Organization showed that road traffic injuries accounted for approximately 262,000 child deaths among children and youth aged 0–19 years—almost 30% of all injury deaths among children[3]. In China, road traffic injuries are the leading cause of death in children under age 14. In 2012 alone, 18,500 children under age 14 died in motor vehicle crashes[4]. Although the number of motor vehicles in China is less than half of that in the United States, motor vehicle crash related deaths among children in China are 3 times the rate in the United States[5]. Of all the road users who died in traffic crashes in China from 2000 to 2008, passengers accounted for 24.3%, second only to pedestrians[6]. Child passengers are a vulnerable population, with high risk of crash-related injuries and deaths[1].

The proper use of child restraint seats reduce risk of fatality among infants by 71% and among toddlers by 54%[7]. Several existing studies on child passenger safety conducted in China showed that usage of child passenger restraint was very low [8–10]. A survey study conducted in Shanghai found that of 970 child passengers aged 3 to 7 years, 60.8% were not restrained by child safety seats (CSS) or booster seats, and 32.0% were sitting on an adult’s lap[8]. The researchers confirmed similar findings in their observation study conducted in Shantou in 2013[10]. Of 3,333 child passengers observed, only 22 (0.6%) used CSS or booster seats, and for infants, toddlers, and preschool children, more than 95% were not placed in child restraints[10].

In more recent years, efforts have been made to improve child passenger safety in China[11,12]. These include targeting birthing mothers in the maternity wards to educate them about the importance of CSS use[11], and providing the parents of children ages 3 to 8 with free CSS to increase use[12]. Despite of these efforts, CSS use rate is still very low. One of the reasons for such low usage is lack of legislation. In the entire country of China, there were no laws or regulations on child passenger safety until March 2014, when Shanghai passed local child passenger safety legislation. The new legislation requires that child passengers younger than age 4 be restrained by CSS and that child passengers younger than age 12 must sit in the back seat[13]. Following the enactment of legislation in Shanghai, several other provinces and cities in China, including Shandong province[14] and Shenzhen city[15] have also passed and enacted local legislation. However, currently, there is no national policy or legislation in China that specifies how to safely transport newborn babies from the hospital to home. Most of the infants leave the hospital unrestrained[11]. Hospitals in China do not bear any responsibility if there are injuries to the newborn on the way home due to not restraining the child in a CSS. Thus, it becomes parents’ responsibility to protect their newborns and ensure safe transportation.

Pregnant women are required to make a series of decisions regarding child safety even before the baby is born. One of the first decisions these expectant mothers need to make is how to ride home safely from the hospital after giving birth. In China, about 50,000 infants are born every day, with the vast majority of them born in hospitals. Understanding of the mothers’ and/or expectant mothers’ concerns regarding child passenger safety, and factors influencing their intended and actual use of CSS is an important first step in protecting children from traffic related injuries and deaths. In this study, we investigated the knowledge, attitudes and behavioral intention regarding use of CSS among parents of newborns, using a quantitative approach. We also explored expectant mothers’ views and decisions regarding CSS use, using a qualitative approach. It is our hope that the knowledge gained from this study will help in the development of interventions targeting expectant mothers and/or parents of newborns to increase their knowledge, intention and behaviors associated with protecting their child passenger from traffic related injuries and deaths.

## Methods

**Fig 1:**
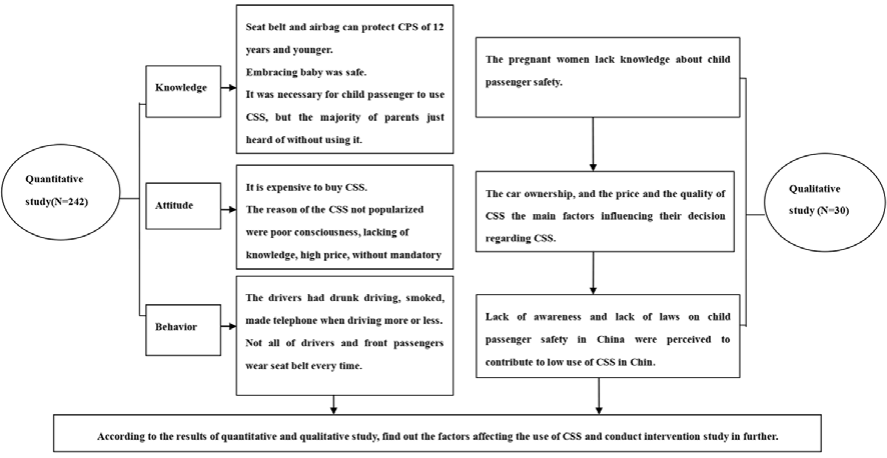
Chart of the Study Procedure.

Both quantitative surveys and qualitative interviews were used in this study to understand the factors influencing the expectant mothers’ decision of use of CSS and the factors influencing the parents’ use of CSS. A quantitative survey was utilized to investigate the knowledge, attitudes, and behaviors on child passenger safety among parents of newborns, while a qualitative interview among expectant mothers was used to help better understand and explain the quantitative results. A detailed description of the study procedure is shown in Fig 1. The study protocol, along with the consent process, was approved by Medical Ethics Committees of Shantou University Medical College.

### Quantitative Survey

#### Survey participants

Two hospitals in Shantou, a coastal city located in China, were randomly selected: (1) Shantou Women’s and Children’s Hospital, a general hospital equipped with 60 ward beds with about 300 infants delivered per month, and (2) Shantou University-the First Affiliated Hospital, a general hospital equipped with 50 ward beds with 250 infants delivered per month. Both were non-profit hospitals. Pregnant women from these two hospitals have similar demographic characteristics, such as language, income, educational background and occupation (P>0.05), and thus, they may share similar views on CSS use. A convenience sample of parents of newborns was recruited to complete a questionnaire in person in the maternity wards after delivery. The inclusion criteria were: (1) the parent of a newborn; (2) both the parent and the newborn were healthy without any adverse postpartum symptoms; and (3) agreed to participate through a signed consent document. Parents who did not speak mandarin or could not read or write were excluded. In the study, no one was excluded because they did not meet the criteria.

#### Survey instrument

The survey instrument was developed by the research team based on the published work and team’s previous work in this area[10]. The questions asked included four parts: Socio-demographic characteristics; knowledge; attitudes; and behaviors about child passenger safety. The reliability and construct validity of the survey instrument was 0.73 and 0.78 respectively.

#### Survey data collection

Four trained medical graduates conducted the survey at two participating hospitals’ maternity wards after delivery. Following the approval of the two hospitals, the head nurse of the maternity wards in the respective hospitals received training on the study protocols. The head nurse informed all the parents of newborns about the research project. Parents who expressed interest in participation were introduced to the research staff. During the meeting, research staff confirmed the parents’ interest, answered any questions the parents had, obtained informed consent, and administered the paper survey. The data collection was conducted between October 2014 and April 2015.

The returned surveys were reviewed by two investigators before entering into the computer to ensure quality of data. A total of 58 returned surveys were excluded from analysis, including 55 incomplete surveys, two blank surveys, and one illegible survey.

#### Main outcome measures

Knowledge of child passenger safety was measured by eight questions, asking participants about their awareness of the car seat, airbag, CSS, and other related issues. Attitudes among newborn parents was measured by three questions including their favorability and resources to obtain child safety information, their view of current CSS legislation in China, and reasons and attributable factors of current low use of CSS in China. Behavior of child passenger safety was measured by three items regarding CSS use and four items about their own driving safety behaviors. These included whether the participants intend to use a CSS. would buy a CSS, or would rent a CSS when the option is available; and whether the participants had ever driven after drinking, had ever smoked while driving, had used a cell phone while driving, or had worn seat belt.

#### Survey data analysis

Survey data were entered into EpiData version 3.0 for windows and then analyzed using SPSS version 19.0. All the data was double-entered to ensure quality. The frequencies for all variables were computed. The differences in knowledge and attitudes of parents of newborns across the subgroups were compared. The factors associated with intent and actual behaviors of CSS use were assessed using logistic regression.

### Qualitative Interview

#### Interview participants

Pregnant women who sought prenatal care at Shantou Women’s and Children’s Hospital were recruited for a semi-structured interview. The inclusion criteria were: (1) women were more than 12 weeks pregnant; (2) owned a car; and (3) agreed to participate through signed consent. Pregnant women who were diagnosed with high risk of pregnancy or who were unable to complete more than 15 minutes of an in-person interview were excluded. The targeted sample size was 30 to reach saturation.

#### Interview guide

A semi-structured interview guide was developed based on existing literature, the objective of the research, and the results of the survey. The key questions included (1) what main concerns the pregnant women had regarding child passenger safety; (2) what factors may potentially influence their decision on whether or not to use CSS, and (3) what they believe the reasons are for current low use of CSS in China.

#### Interview data collection

The in-depth interviews were conducted face-to-face by one of the two authors who were trained in qualitative interviewing and study protocols. Before the interview, the pregnant women who expressed interest were informed about the purpose and process of the interview, were screened for eligibility, and signed the informed consent. Each interview was conducted in a private room in the hospital and lasted about 30 minutes. All interviews were audio recorded and transcribed into electronic documents word-for-word on the same day by another author of this manuscript before data analysis.

#### Qualitative data analysis

The transcribed electronic documents were imported into software NVivo 8.0 and read repeatedly by two researchers, who coded and analyzed thematically. Based on the content of the documents, the research team developed unified coding rules. Each coding was discussed by two researchers. If they encountered disagreement in the process of encoding, the researchers discussed and refined codes together until consensus could be achieved. Through this coding process, key words or themes and unified ideas which described the factors that influenced their decision of use of CSS and the reasons of low use of CSS in China were identified as the themes and sub-themes. All of the 30 coding documents were ultimately generated and categorized into three final themes. The data and results were assessed by researchers who speak English, Chaozhou-Shantou dialectal and Mandarin, the languages used by the participants.

## Results

### Characteristics of Parents of Newborns in the Quantitative Survey Study

A total of 300 questionnaires were distributed and 242 valid surveys were returned, with a response rate of 80.7%. The socio-demographic characteristics of the participants are shown in Table 1. Of 242 participating parents, 93 (38.4%) were male and 149 (61.6%) were female, with 165 (68.2%) of participants aged from 21 to 30 years. The average age of the participants was 28.7 (±4.395). Nearly two-thirds (65.7%) of participating parents resided in Shantou. More than two-fifths of them had received undergraduate or higher education (male and female accounted for 44.6% and 43.4% respectively).

**Table 1:** Demographic Characteristics of Surveyed Newborn Parents.

Characteristics		All	Ever used CSS		*χ*^*2*^	*P-*Value
			Yes n(%)	No n(%)		
**Gender**	Male	93	50	43	3.891	0.049
	Female	149	99	50		
**Age**	<20	7	5	2	1.343	0.719
	21–30	165	101	64		
	31–40	67	42	25		
	>41	3	1	2		
**Place of residence**	Shantou	159	99	60	0.094	0.782
	Except Shantou	83	50	33		
**Newborn father’s degree of education**	Primary school or below	8	4	4	4.108	0.122*
	High school	126	71	55		
	University or above	108	74	34		
**Newborn mother’s degree of education**	Primary school or below	9	3	6	6.954	0.031
	High school	128	73	55		
	University or above	105	73	32		
**Newborn father’s profession**	Directors or technicians	86	55	31	8.071	0.045
	Businessman	97	58	29		
	Workers or farmers	44	27	17		
	Unemployed or others	25	9	16		
**Newborn mother’s profession**	Directors or technicians	74	50	24	7.317	0.062
	Businessman	44	30	14		
	Workers or farmers	25	18	7		
	Unemployed or others	99	51	48		
**Household per capita income**	<*¥* 4000	82	51	31	0.020	0.886
	≥*¥*4000	160	98	62		
**Whether own a car?**	No	48	13	35	79.473	0.000
	Yes	194	136	58		

* Fisher's exact probability method.

### Past Driving-Related Behaviors of Parents of Newborns

**Fig 2:**
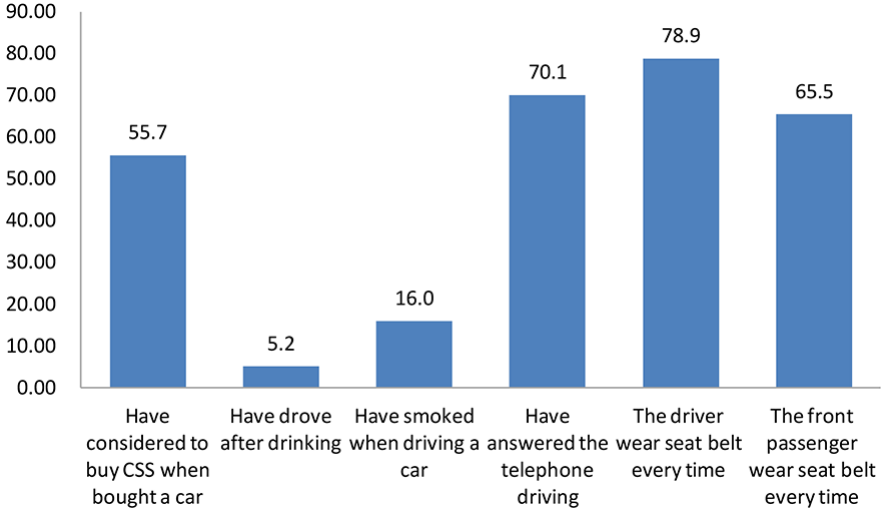
Parents' Behaviors on Driving Safety Percent (%).

Of the total (242) respondents, 194 owned at least one car, 108 (55.7%) considered buying a CSS when they bought their car; 10 (5.2%) reported that they had ever driven after drinking; 31 (16.0%) had ever smoked cigarettes while driving; 136 (70.1%) had answered phone calls while driving. A majority of the participants (n = 153, 78.9%) reported wearing seat belts every time as a driver, but only 127 (65.5%) participants reported wearing seat belts every time they were the front passenger (Fig 2).

### Parental Knowledge of and Attitudes toward Child Passenger Safety

The parental knowledge and attitudes towards child passenger safety are shown in Table 2. Of 242 respondents, 113 (46.7%) parents thought that seat belts could protect child passengers who were 12 years and younger, and 135 (55.8%) parents thought airbags could protect child passengers aged 12 years and younger. Nearly one-fourth (n = 60, 24.8%) of parents believed holding their baby on the lap was safer. A majority of (83.5%) respondents had heard of CSS, and 168 (83.2%) thought it was necessary to use CSS in order protect child passengers. When asking about their preferred methods and channels to receive child passenger safety information, 57.8% of parents indicated that they preferred to receive brochures, while 22.3% preferred to search for such information by themselves. Nearly three quarters of respondents (n = 147, 72.8%) indicated that the present price of CSS on the market was high, and only 7 (3.5%) participants thought the CCS price was very affordable. When asking about the reasons why CSS was not popular in China, the participants indicated that poor awareness and knowledge of child passenger safety was the top reason (78.7%), followed by high price (10.9%), and a lack of mandatory national legislation (8.9%); the other reasons included inconvenience to use, occupying too much interior space, and having a car that cannot have a CSS installed.

**Table 2:** Attitudes and Knowledge on CPS of Surveyed Newborn Parents.

Factors		The usage of CSS		*χ*^*2*^	*P-*Value
		Use n(%)	Non-use n(%)		
**Whether the car seat belt can protect the child passenger safety under the age of 12 or not?**	Yes	65	48	1.468	0.236
	No	84	45		
**Whether the car airbag can protect the child passenger safety under the age of 12 or not?**	Yes	77	58	2.652	0.103
	No	72	35		
**Whether holding baby on the lap is safe or not?**					
Yes	Yes	33	27	1.456	0.284
	No	116	66		
**Whether letting child alone in a car is safe or not?**	Yes	1	0	0.627	1.000*
	No	148	93		
**Whether feeding child in a driving car is safe or not?**	Yes	17	3	5.058	0.025
	No	132	90		
**Have you ever heard of CSS?**	Yes	136	66	17.115	0.000
	No	13	27		
**How is the price of CSS?**	Expensive	103	44	4.255	0.119*
	Cheap	1	6		
	Unclear	21	27		
**Whether it is necessary for child passenger to use CSS?**	Yes	117	51	2.485	0.289
	No	7	5		
	Unclear	12	10		
**What the reason of low use of CSS?**	Poor knowledge and safety awareness	109	50	8.069	0.045*
	No laws and regulations	8	10		
	Too expensive	18	4		
	Other reasons	1	2		

* Fisher's exact probability method.

### Factors Influencing CSS Use

Use of CSS was statistically significantly higher among participants who were female, who were fathers with undergraduate or higher level of education, who were not stay at home mothers, and who owned a car (Table 1). Additionally, participants who had not heard about CSS before, or who believed that feeding a child in a driving car was safe had low use of CSS. After adjusting for gender, household income per capita, education level, occupation, car ownership, and knowledge of child passenger safety, results from multivariable logistic regression analysis showed that occupation of father, occupation of mother, household income per capita, car ownership, and knowledge of child passenger safety were significantly associated with CSS use (Table 3).

**Table 3:** Adjusted odds ratios of intended use of CSS among parents of newborn^a^.

Factors		*OR*	*95%CI*		*P-*value
**Gender**	Male	1			
	Female	1.142	0.530	2.462	0.735
**Newborn mother’s degree of education**	Primary school or below	1			0.870
	High school	0.888	0.133	5.928	0.903
	University or above	1.224	0.516	2.905	0.646
**Newborn father’s profession**	Directors or technicians	1			0.071
	Businessman	4.673	1.283	17.041	0.019
	Workers or farmers	4.041	1.113	14.679	0.034
	Unemployed or others	6.395	1.429	28.615	0.015
**Newborn mother’s profession**	Directors or technicians	1			0.196
	Businessman	1.358	0.518	3.560	0.534
	Workers or farmers	1.572	0.547	4.519	0.401
	Unemployed or others	4.513	1.120	18.179	0.034
**Household per capita income**	<*¥* 4000	1			
	≥*¥*4000	0.424	0.180	0.997	0.049
**Whether own a car?**	No	1			
	Yes	29.448	10.986	78.931	0.000
**Whether feeding child in a driving car is safe or not?**	Yes	1			
	No	0.197	0.039	0.999	0.050
**Have you ever heard of child safety seat?**	No	1			
	Yes	2.347	0.819	6.727	0.112
**Constant**		0.037			0.039

^a^ Multivariable logistic regression adopt stepwise regression variable model, entry of 0.05, removal of 0.10

### Characteristics of Pregnant Women in the Qualitative Study

Of 30 pregnant women interviewed, 23 (76.7%) were pregnant for the first time while 7 (23.3%) were in their secondary pregnancy, with ages ranging from 22 to 40. Twenty-five of the 30 participants were native to Shantou, and the rest were originally from other parts of China and were working and living in Shantou during the study. All participants owned at least one private car, and four of them drove the car by themselves, while the rest rode in a car with their husbands to and from work daily.

### Three Key Themes Identified

The three key themes identified from interview data were: (1) the pregnant women lack knowledge about child passenger safety; (2) the car ownership, and the price and the quality of CSS the main factors influencing their decision regarding CSS use, and (3) lack of awareness and lack of laws on child passenger safety in China were perceived to contribute to low use of CSS in China.

The sub-themes of parents’ experiences and practices related to child passenger safety were derived from the three major themes. Respective supporting quotes were:

1. Parents wrongly believed that holding a child on his/her lap was safer than restraint by CSS.
  *I feel that holding children on laps is safer*. *It is a natural human response that people would hold children tightly when a car brakes*. *If a child is restrained in a safety seat*, *I feel I could not protect my child when a car suddenly brakes*.
  ———A pregnant woman who had held her child passenger
2. Although they had seen it, parents don't know how to select and install CSS, nor the price of CSS.
  *I think the CSS is quite necessary*. *Especially when the unplanned hard brakes or traffic accidents happen*, *it acts as a buffer*. *The child is likely to be left out without the buffer of an installed device*. *Having not used CSS*, *I don't know how much it costs*. *Of course*, *I don’t know how to install it either*.
  ———A pregnant woman who didn’t use CSS
3. Mandatory legislation can effectively improve the utilization rate of CSS.
  *The mainly reason is the legal vacancy*. *For example*, *new laws on front seat passengers indicate that sitting in the front seat without wearing a seat belt will be punished 2 points and fined 200 Chinese Yuan*. *With enactment of such laws*, *everybody is required to wear seatbelts*. *So to improve CSS use*, *new laws on child passenger safety are urgently needed*.
  ———A pregnant woman who drove her car to work everyday

We found that parents with poor awareness of child passenger safety often exhibited risk behaviors, including holding or feeding a baby in a moving car. To protect child passengers, parents must be targeted for their awareness of child passenger safety. The results from this study indicate that the major factors that influence parents’ decision of CSS use were whether they own a car, and the price and quality of CSS. In addition, a lack of mandatory legislation was an important factor contributing to the low CSS use in China. These findings were confirmed through both quantitative and qualitative approaches, and provide the foundation for a future intervention focused on reducing child passenger injuries.

## Discussion

While there is a body of literature on child passenger safety using quantitative approaches[1,5,10,11,16–19], little has been done using qualitative approaches[20,21]. Existing studies have little been specifically focused on expectant mothers[21,22]. This is the first study conducted in China examining pregnant women’s attitudes and behavioral intention regarding CSS use. Focusing on pregnant women as a potential target population for child passenger safety has several advantages. First, as prospective parents, pregnant women, especially those who are pregnant for the first time, desire to stay close to the child for taking care of them[21]. Second, as the mothers to be, they are more like to be trained to practice child passenger safety behaviors[23]. Finally, most pregnant women go to the hospital for regular prenatal care, so implementing child passenger safety education program during their visit has practical value[21].

Our findings showed that the parental knowledge on child passenger safety was poor, and that many parents did not have a good understanding of child restraint systems. Specifically, 46.7% of parents of newborns considered seat belts to protect child passengers aged 12 years and younger, and over half (55.8%) of parents of newborns thought that airbags could protect child passengers. Research has demonstrated that seat belts are a restraint system for adults, and that children’s bodies are too small to be protected effectively[12]. Studies have shown that child passengers aged 5 to 9 who use an adult seat belt have an incidence of injuries that is 2.7 times that of adults[8]. Airbags are also an important contributing factor to child's head and neck damage, and the enormous impulse force at the moment of expansion can be fatal to a child passenger. Even in the case of equipping a car with a child restraint system, the child passenger should also be far away from the airbag.

The parents’ poor awareness of child passenger safety is one of the major contributing factors to the child passenger related risk behaviors. Over 80% of respondents had heard of CSS, and among them, 48.3% indicated it was necessary for child passengers to use CSS. During the interviews, we also learned that a majority of pregnant woman had seen CSS on television or at a CSS store, and thought it had a protective effect on child, but without using it, they didn’t know how to install it. While all of the parent participants in our quantitative survey study owned private cars, 63.2% of them had considered purchasing a CSS. However, actual purchase and use of CSS was rare. A recent study showed that 75.6% of child passengers didn’t use CSS in China[24]. Previous studies conducted in China showed that the rate of CSS use was about 5% in Beijing[9] and in Shanghai[8], both metropolises with a large number of private cars. In the small city of Shantou, it was less than1%[10].

Consistent with previous study findings[11], public education on child passenger safety was also identified as one of two effective approaches to prompt CSS use in our study. Nearly 80% (78.7%) of parents in our survey thought the reasons that CSS use was not popular in China was people's poor consciousness coupled with a lack of knowledge. Thus, designing and implementing programs that focus on the improvement of parents’ knowledge of and attitudes toward child passenger safety might be the most important first step to help increase CSS use. These programs would be particularly practical in a small city like Shantou, where the economic development of the city makes it challenging to immediately enact a law requiring every child passenger to use CSS. These programs should involve the efforts from the government, education department, hospitals, and CSS enterprises[11,25,26]. These programs could be delivered through TV, news broadcast, and other media, as many of our interview participants indicated that they gained knowledge of child passenger safety through these channels. Another effective strategy to improve CSS use is to have mandatory use legislation.

In our survey study, nearly 10% of respondents thought no mandatory national legislation was another reason for low CSS use in China, which was supported by the results of our interview study. Mandatory legislation has been shown to effectively improve the utilization rate of CSS in other countries[10,25,27,28]. However, national legislation is unlikely to be put in place soon in China, although Shanghai and Shandong provinces have implemented local regulations, which require child passengers less than age 4 to use CSS. In the United States, after the legislation enforcement, the CSS utilization rate increased by 6% per capita, and child road traffic injuries decreased by 5% per capita[27]. Some researchers believe that the current knowledge level of CSS in Chinese parents is equivalent to the level of western countries in 2000[8], although traditionally, Chinese parents pay more attention to their children. Our findings, along with others, call for additional efforts on enactment of the laws and legislation to effectively increase the use of CSS and reduce road traffic injuries[1,28].

This study has three limitations. First, the data on attitudes, and intended and actual use of CSS use was based on self-report, thus respondents may not report truthfully due to social desirability. Second, there was a selection bias. Both the qualitative and quantitative study used convenience sampling to recruit subjects. Thus, the participants may not represent all expecting mothers and parents of newborns. Third, the sample in the quantitative research was small in size. As new mothers need to rest after delivery, the father of newborn is busy taking care of the infant, the mother, and visiting friends and relatives. Even though we made every effort to recruit fathers, we were not able to recruit a large sample. Finally, because of the nature and purpose of this study, advanced statistical methods were not used to control for confounding factors when assessing knowledge, attitudes, and intended behaviors about use of child safety seats among parents of newborns. Further research with larger sample sizes and more comprehensive analytic approaches are warranted.

## Conclusions

This study documented the knowledge, attitudes and behaviors towards child passenger safety among parents of newborns. Participants had a poor awareness of child passenger safety with low risk perception. Effective measures must be taken to improve parents’ knowledge and perceptions with child passenger safety promotion before legislative interventions are implemented.

## Supporting Information

S1 File S1 File. (XLS)
